# Supplementary figures and images for: The Dynamic Transition of Persistence toward the Viable but Nonculturable State during Stationary Phase Is Driven by Protein Aggregation
Source: mBio. 2021 Aug 3;12(4):e00703-21. doi: 10.1128/mBio.00703-21 (PMC8406143; doi:10.1128/mBio.00703-21)

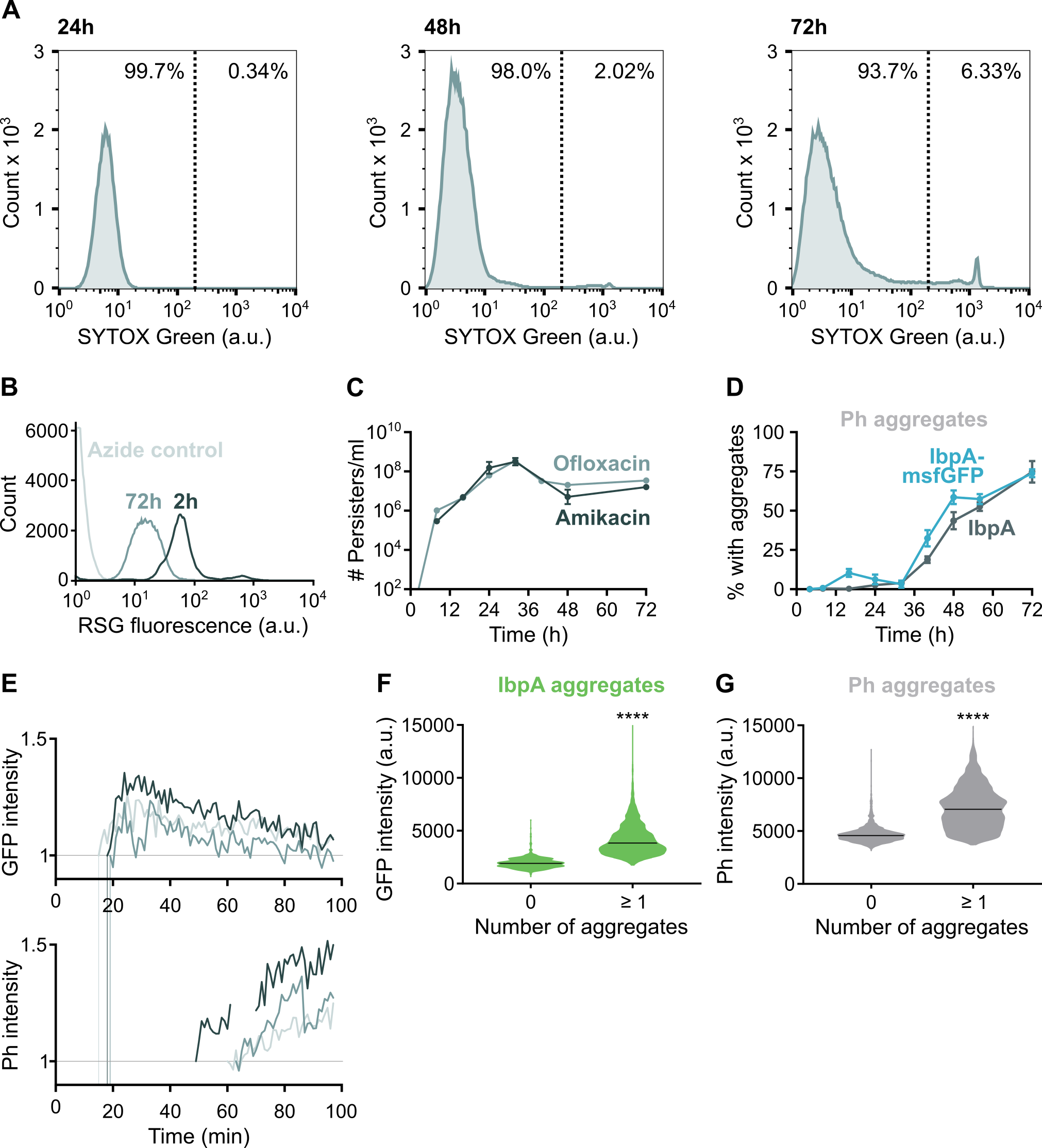

Supplement: FIG S1 [file mbio.00703-21-sf001.tif]

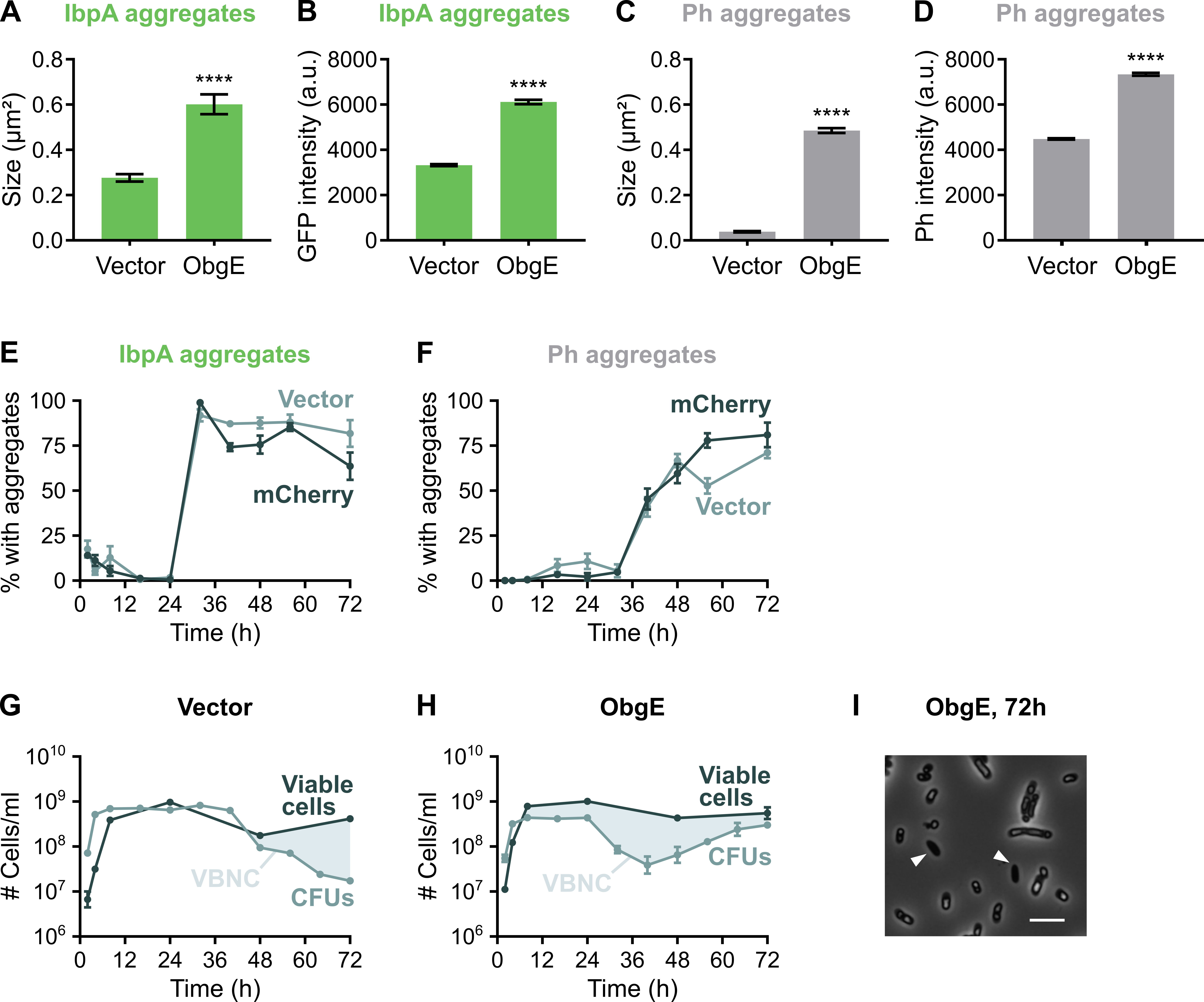

Supplement: FIG S2 [file mbio.00703-21-sf002.tif]

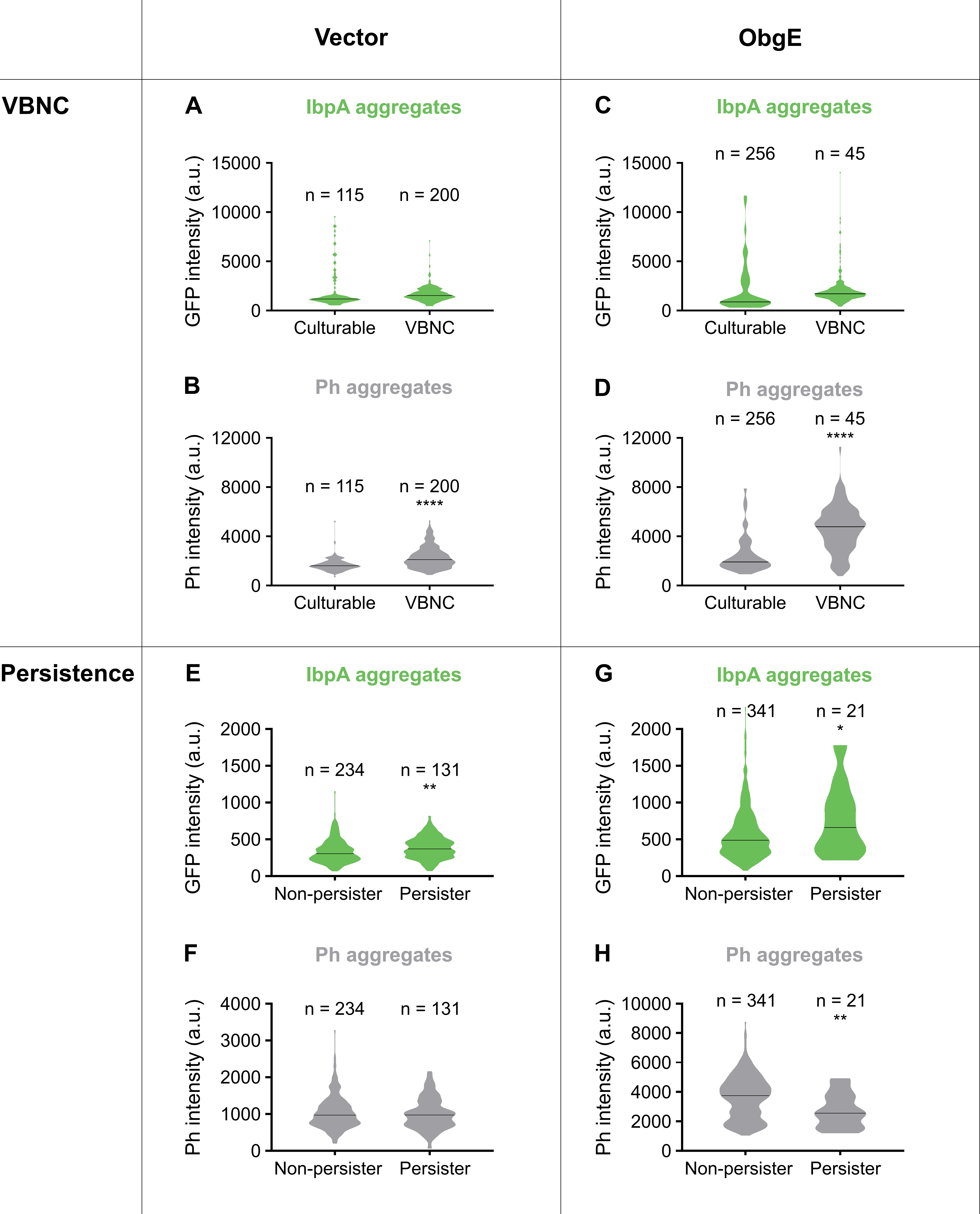

Supplement: FIG S3 [file mbio.00703-21-sf003.tif]

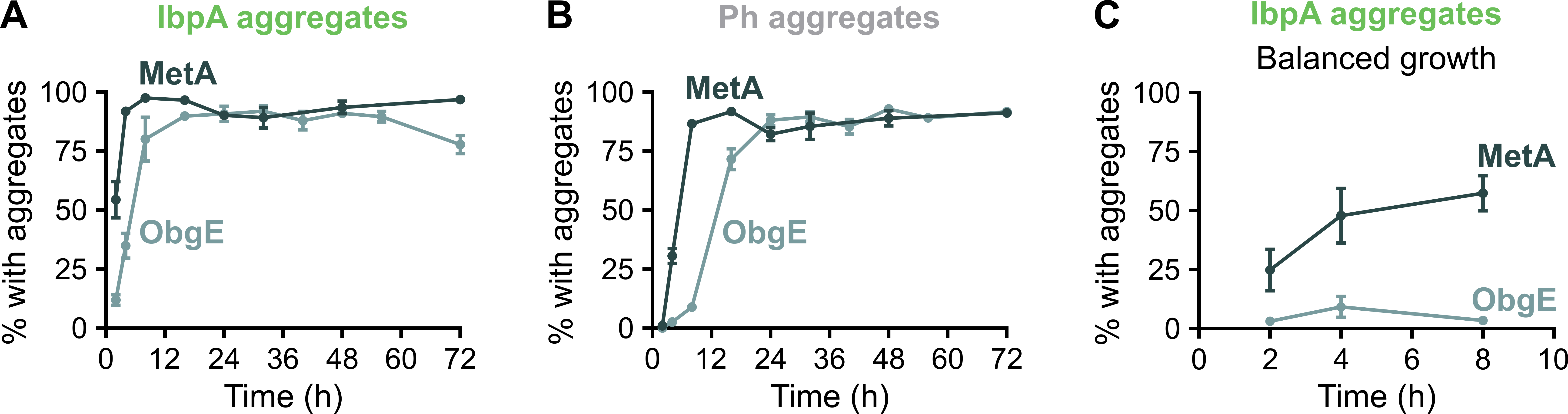

Supplement: FIG S4 [file mbio.00703-21-sf004.tif]

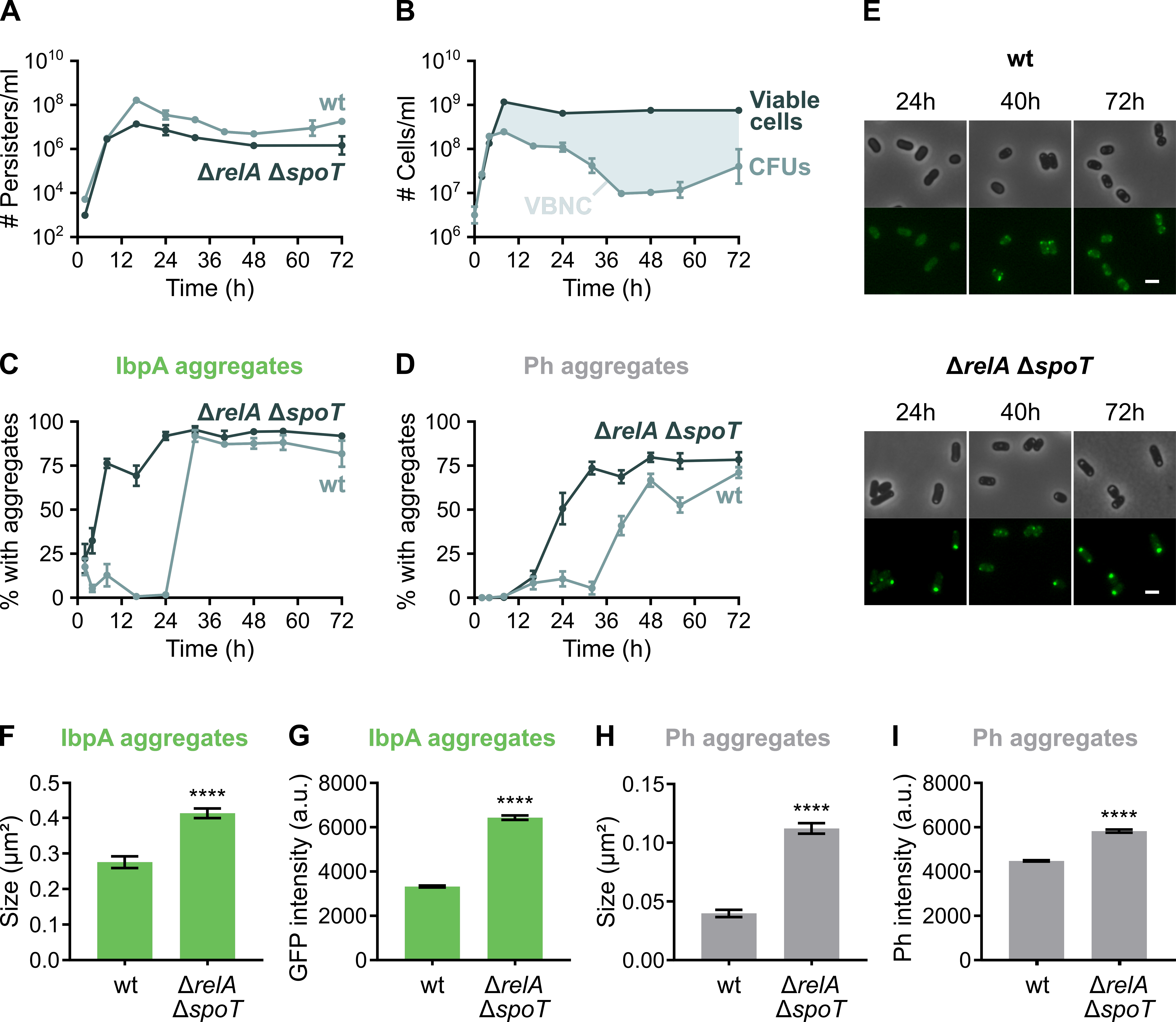

Supplement: FIG S5 [file mbio.00703-21-sf005.tif]

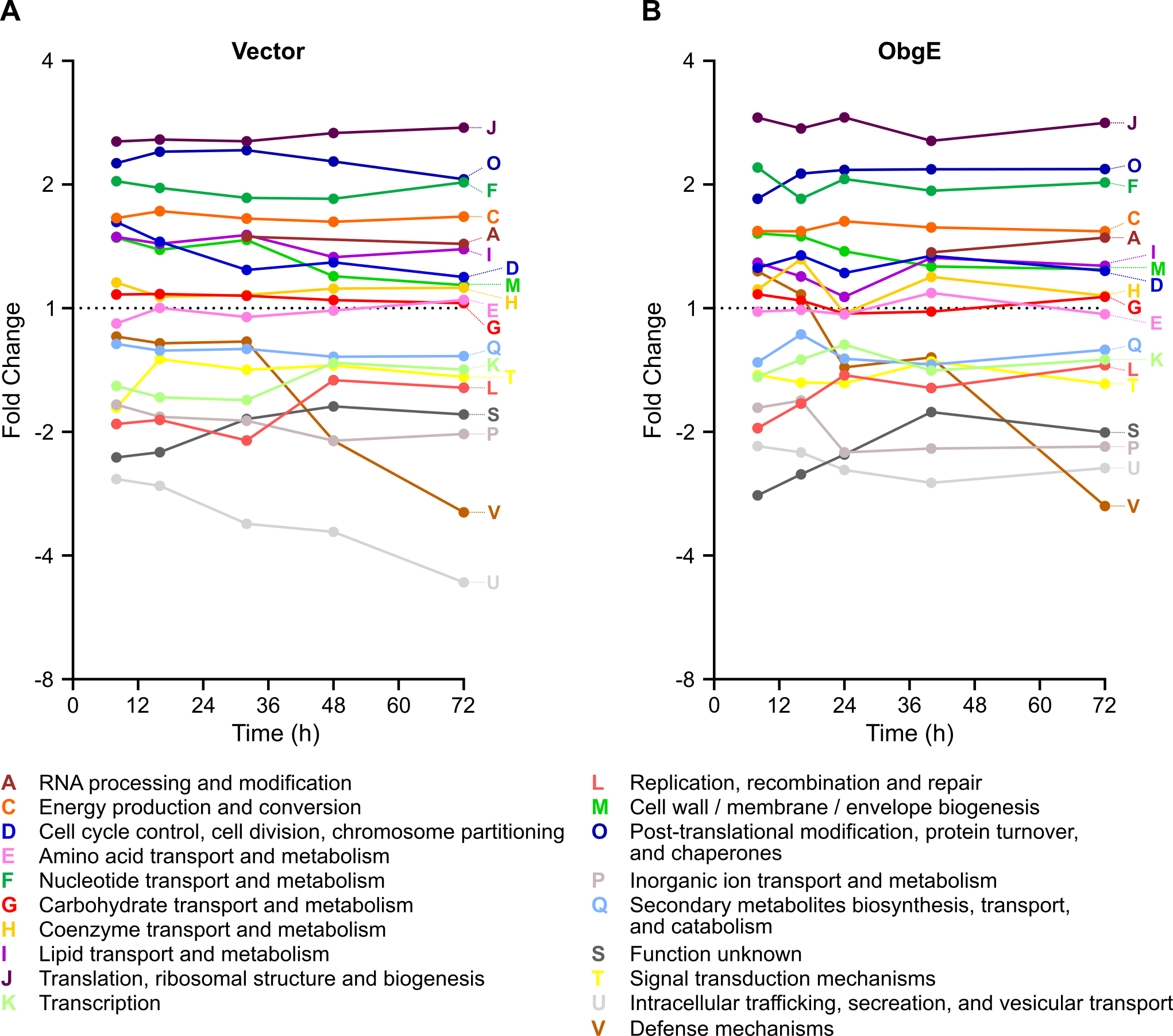

Supplement: FIG S6 [file mbio.00703-21-sf006.tif]
